# A zebrafish model of *acmsd* deficiency does not support a prominent role for ACMSD in Parkinson’s disease

**DOI:** 10.1038/s41531-025-00940-1

**Published:** 2025-05-09

**Authors:** Emma Fargher, Marcus Keatinge, Oluwaseyi Pearce, Petteri Piepponen, Pertti Panula, Fredericus J. M. van Eeden, Ryan B. MacDonald, Oliver Bandmann

**Affiliations:** 1 Sheffield Institute for Translational Neuroscience (SITraN), University of Sheffield, Sheffield, UK; 2 Bateson Centre, Firth Court, University of Sheffield, Sheffield, UK; 3 Division of Pharmacology and Pharmacotherapy, University of Helsinki, Helsinki, Finland; 4 Department of Anatomy, University of Helsinki, Helsinki, Finland; 5 Present Address: Centre for Discovery Brain Sciences, Chancellor’s Building, The University of Edinburgh, Edinburgh, UK; 6 Present Address: Institute of Ophthalmology, University College London, London, UK

**Keywords:** Genotype, Mutation, Animal disease models, Genetic models, Neurological models, Biological models, Experimental organisms, Imaging, Metabolomics, Microscopy, Parkinson's disease, Diseases of the nervous system, Parkinson's disease, Biological techniques, Genetics, Neuroscience, Medical research, Neurology, Pathogenesis, Risk factors, Diseases, Neurological disorders, Movement disorders, Parkinson's disease, Experimental organisms, Model invertebrates, Transgenic organisms

## Abstract

Single nucleotide polymorphisms adjacent to the α-amino-β-carboxymuconate-ε-semialdehyde decarboxylase (ACMSD) gene have been associated with Parkinson’s disease (PD) in genome-wide association studies (GWAS). However, its biological validation as a PD risk gene has been hampered by the lack of available models. Using CRISPR/Cas9, we generated a zebrafish model of *acmsd* deficiency with marked increase in quinolinic acid. Despite this, *acmsd*^*-/-*^ zebrafish were viable, fertile, morphologically normal and demonstrated no abnormalities in spontaneous movement. In contrast to the postulated pro-immune pathomechanism linking ACMSD to PD, microglial cells and expression of the proinflammatory cytokines *cxcl8, il-1β*, and *mmp9* were similar between *acmsd*^*-/-*^ and controls. The number of ascending dopaminergic neurons, and their susceptibility to MPP+, was also indistinguishable. An upregulation of kynurenine aminotransferase activity was identified in *acmsd*^*-/-*^ zebrafish which may explain the absence of neurodegenerative phenotypes. Our study highlights the importance of biological validation for putative GWAS hits in suitable model systems.

## Introduction

Parkinson’s disease (PD) is a common, progressive neurodegenerative disorder with a loss of dopaminergic neurons in the substantia nigra as its pathological hallmark^1^. Accumulating evidence suggests an important role for neuroinflammation in PD pathogenesis^2^. Genome-wide association studies (GWAS) have identified over 90 genetic risk variants for sporadic PD^3^, with many single nucleotide polymorphisms (SNPs) occurring in or nearby genes expressed in immune cells or otherwise linked to inflammation.

**Fig. 1:**
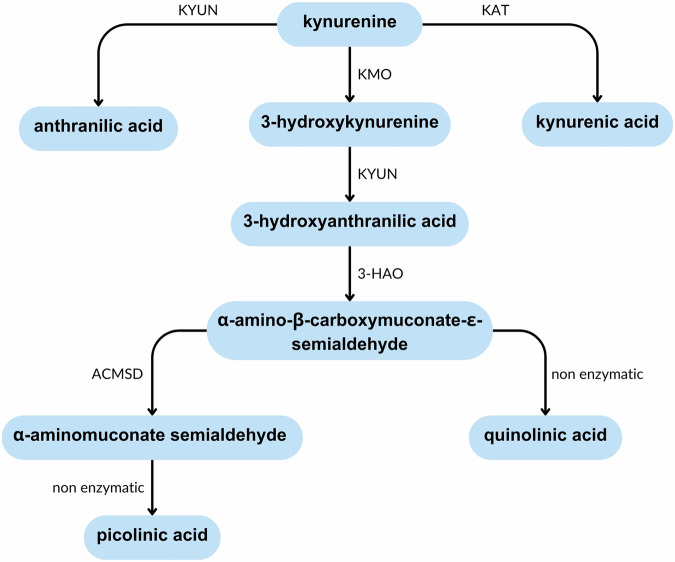
Summary of the kynurenine pathway. The kynurenine pathway is involved in the catabolism of tryptophan. The production of picolinic acid is considered an anti-inflammatory branch of the pathway, whist the production of quinolinic acid is considered pro-inflammatory due to its ability to activate immune cells and induce cell death. ACMSD acts at a branching point in this pathway that determines which of these products is created. 3-HAO 3-hydroxyanthranilate oxidase, ACMSD α-amino-β-carboxymuconate-ε-semialdehyde decarboxylase, KAT kynurenine aminotransferase, KMO kynurenine-3-monooxygenase, KYUN kynureninase.

*α-amino-β-carboxymuconate-ε-semialdehyde decarboxylase (ACMSD*) lies under a GWAS peak on chromosome 2^4^, suggesting a possible role for *ACMSD* in the pathogenesis of PD. At least four SNPs in close proximity to *ACMSD* have demonstrated a significant association with PD, although this appears to vary by population^4–11^. The gene expression database GTEx Portal (gtexportal.org) suggests that *ACMSD* expression occurs primarily in the liver and kidneys in humans. However, expression has also been demonstrated at lower levels in the brain^12^. ACMSD enzymatically converts α-amino-β-carboxymuconate-ε-semialdehyde (ACMS) to α-aminomuconate semialdehyde (AMS) at a critical branching point in the catabolism of tryptophan via the kynurenine pathway (Fig. 1). In the presence of ACMSD, the kynurenine pathway terminates with the production of a neuroprotective, anti-inflammatory molecule named picolinic acid (PIC). However, in the absence of ACMSD, ACMS will dehydrate to form quinolinic acid (QUIN), a potent neurotoxin. Its neurotoxic ability is conferred via a number of mechanisms, in particular excitotoxicity via the direct activation of NMDA receptors^13,14^ and by increasing the release, and inhibiting the reuptake and degradation, of glutamate in synaptic regions^15–18^. QUIN also induces neuroinflammation. Whilst microglia and macrophages are the primary source of QUIN in the CNS, they also respond to pathological levels. QUIN directly activates astrocytes and microglia, resulting in NF-κB pathway activation and increased secretion of proinflammatory mediators, such as IL-1β, TNF-α, and COX-2, which in turn can induce neuronal cell death^19–23^. Pharmacological inhibition of the NMDA receptor is unable to prevent this activation, suggesting that it occurs via an NMDA receptor-independent mechanism^24^. QUIN also generates a local proinflammatory environment by enhancing oxidative stress in microglia, astrocytes and neurons^19,25–28^. Interestingly, the inhibition of microglial activation reduces QUIN-induced neuronal toxicity^23^. These data suggest that the inflammatory effect of QUIN plays an important role in QUIN-induced neurotoxicity, as opposed to direct NMDA receptor activation alone. QUIN may also have other, less well studied toxic effects including mitochondrial dysfunction^19^, cytoskeletal disruptions^29^, and disruption of the blood-brain barrier^30,31^.

Zebrafish represent an excellent vertebrate model organism for studying gene function and neurodegenerative disease, exhibiting neurodegenerative markers such as neuronal loss and microglia activation, even during larval stages^32–38^. Zebrafish have been widely used by us and others to biologically validate genes implicated in PD, including *lrrk2*, *gch1, gba1, pink1, dj-1,* and *parkin*^32–34,39–45^.

The objective of this study was to determine the biological effect of *acmsd* deficiency on PD-relevant mechanisms in *acmsd*^*-/-*^ mutant zebrafish, the first vertebrate model of *ACMSD* deficiency.

## Results

### *acmsd*^-/-^ mutation was loss-of-function in zebrafish

**Fig. 2:**
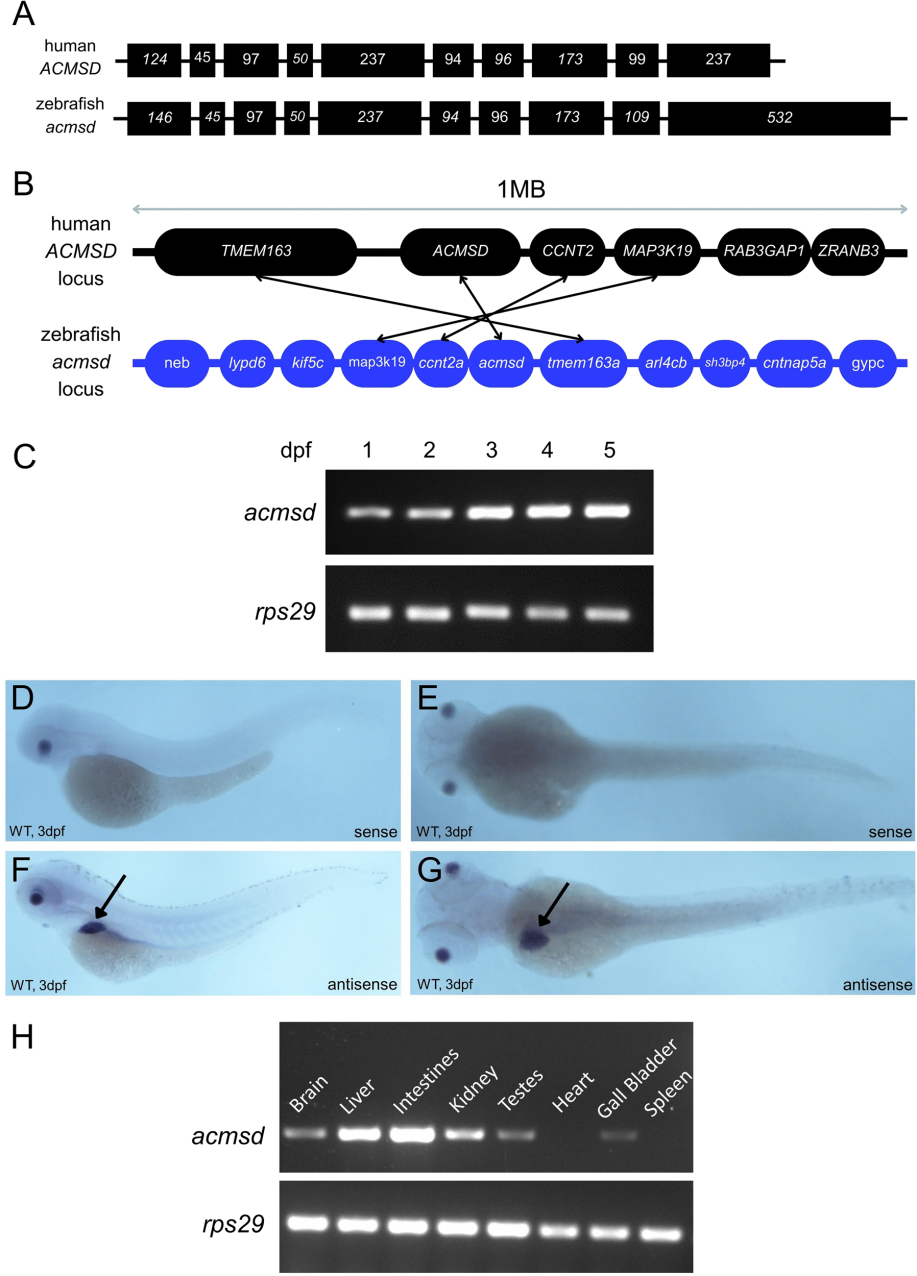
*acmsd* is expressed primarily in the liver and intestines of wildtype zebrafish. **A** Exonic structure of *ACMSD*. Human *ACMSD* (ENSG00000153086) has a single orthologue in zebrafish (ENSDARG00000062549) which shares 81% sequence homology with its human counterpart (based on CLUSTAL W v1.81 data). **B** Synteny has been retained between species. The region comparison feature on Ensembl was used to compare a 1 Mb region around the Acmsd gene on human chromosome 2 and zebrafish chromosome 9. Not all genes in the region are included. Orthologous genes are identified by arrows. **C** RT-PCR demonstrated *acmsd* expression with an increasing concentration from 1 to 5dpf in *acmsd*^*+/+*^ larvae. In situ hybridisation demonstrated restricted *acmsd* expression to the liver (arrows) and gut at 3dpf, shown from lateral (**F**) and dorsal (**G**) views. The sense probe produced no staining (**D**, **E**). **H** RT-PCR demonstrated *acmsd* expression primarily in the liver and intestines of both adult male (shown) and adult female (not shown) zebrafish. Expression was also identified in the brain, kidney, gonad,s and gall bladder.

**Fig. 3:**
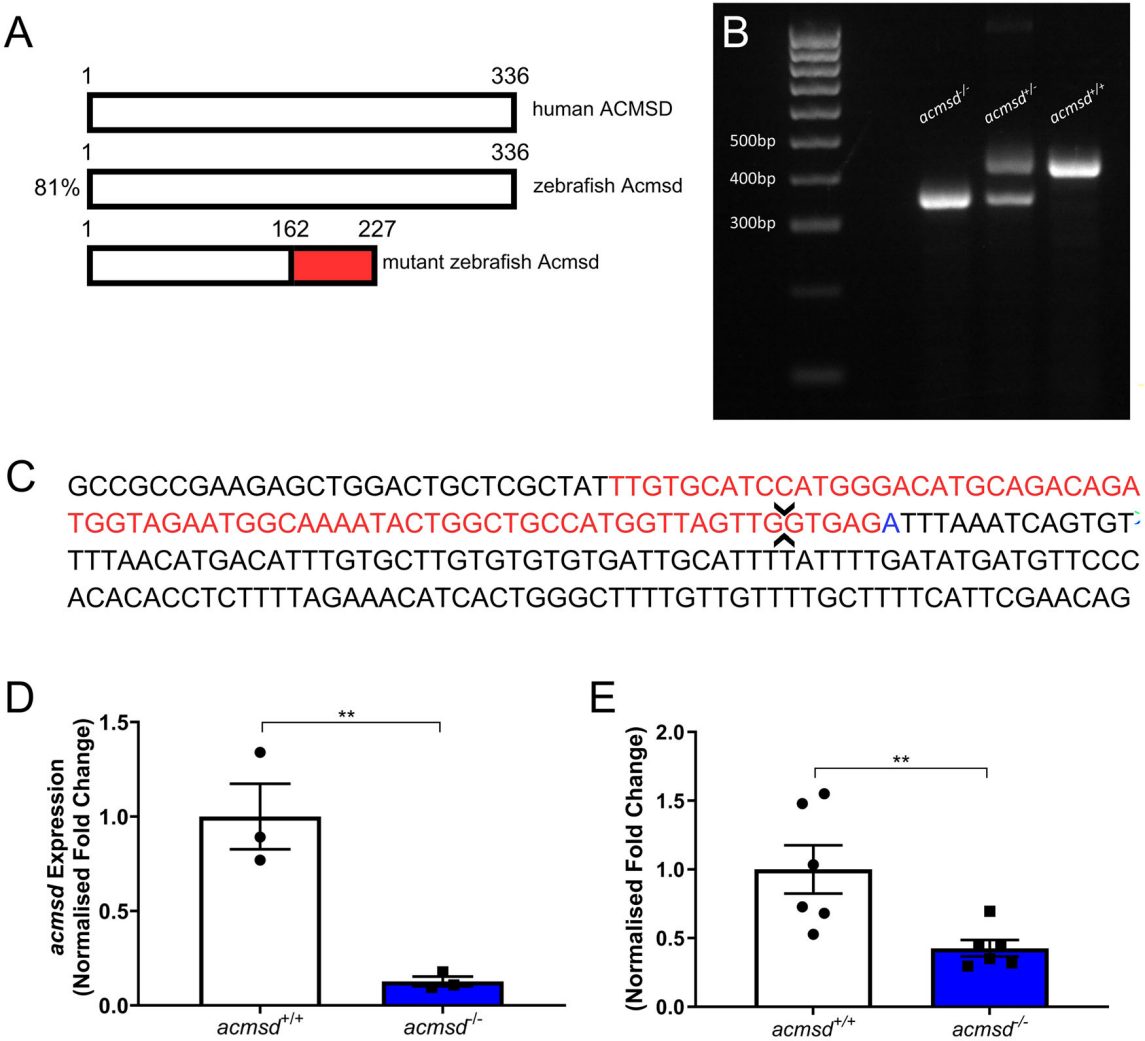
CRIPSR-generated mutation in *acmsd* causes nonsense-mediated decay of resulting mRNA. **A** Zebrafish express a single Acmsd protein, sharing 81% protein identity to human ACMSD. A mutation was introduced into exon 6 using CRISPR/Cas9 resulting in a predicted truncated protein. Red region depicts altered amino acids in the mutated protein. **B** The resulting 70 bp reduction in DNA length allowed for genotyping by standard PCR. *acmsd*^*+/+*^ zebrafish were identified by a single 425 bp band (right), homozygous *acmsd*^*-/-*^ mutants by a 355 bp band (left), and heterozygous *acmsd*^*+/-*^ mutants by a double band (centre). **C** DNA sequence of *acmsd* exon 6 and intron 6. *acmsd*^*-/-*^ zebrafish possessed a 71 bp deletion (red) and 1 bp insertion (blue) in this region, resulting in the loss of a 3’ splice site (arrows). *acmsd*^*-/-*^ larvae demonstrated reduced *acmsd* expression compared to their *acmsd*^*+/+*^ siblings at 5dpf (**D**, *n* = 3 biological replicates (15 larvae per replicate), *p* = 0.0012) and in adult brain tissue (**E**, *n* = 6 biological replicates (1 brain per replicate), *p* = 0.0040). Statistics from two-tailed *t* tests using ddCt values.

Human *ACMSD* has a single orthologue in zebrafish, *acmsd*, which demonstrates ~80% DNA and protein homology (Figs. 2A, 3A) and conserved gene synteny between species (Fig. 2B). Using RT-PCR, we found that *acmsd* was expressed at increasing levels from 1 to 5dpf in whole larvae (Fig. 2C). In situ hybridisation demonstrated that this expression was largely limited to the liver, although *acmsd* staining was also observed in intestinal tissue (Fig. 2D–G). RT-PCR based studies demonstrated more widespread expression in adult zebrafish, with the liver and intestines demonstrating the highest expression. Lower *acmsd* expression levels were identified in the brain, kidney, gonads, and gall bladder (Fig. 2H).

To create a stable *acmsd*^*-/-*^ line, we utilised CRISPR/Cas9 technology. The resulting mutant had a 1 bp insertion and 71 bp deletion in exon 6, resulting in the deletion of a 3’ splice site (Fig. 3A–C). Sequencing of homozygous mutant cDNA revealed that exon 6 underwent complete exon skipping without the retention of surrounding introns. Flanking exon sequences were indistinguishable from *acmsd*^*+/+*^ cDNA. In silico transcription suggested that the loss of exon 6 would result in the production of a truncated Acmsd protein (Fig. 3A). Importantly, this mutation induced a premature STOP codon in exon 8 which triggered nonsense-mediated decay of the resulting transcript in both whole larvae and adult brain tissue (Fig. 3D, E). Compared to *acmsd*^*+/+*^, *acmsd* mRNA levels were 87% lower in *acmsd*^*-/-*^ larvae and 58% lower in *acmsd*^*-/-*^ adult brain tissue.

### *acmsd*^*-/-*^ zebrafish do not develop overt neurodegenerative phenotypes

**Fig. 4:**
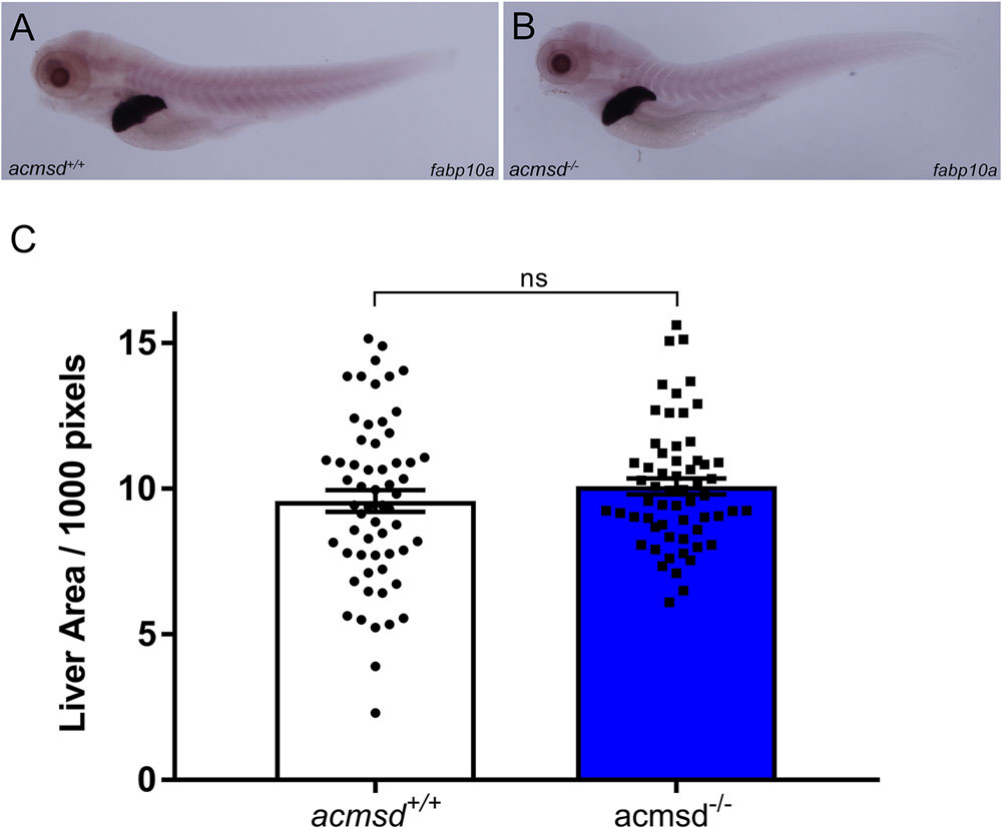
Liver size does not vary between *acmsd*^*+/+*^ and *acmsd*^*-/-*^ larvae. 5dpf zebrafish underwent in situ hybridisation against fatty acid-binding protein 10a *(fabp10a*, **A**, **B**, representative images). **C** There was no difference in liver area identified between genotypes (*p* = 0.2762, unpaired two-tailed *t* test). Data from three biological replicates, *n* = 59 per genotype. Data points represent individual fish.

Despite *acmsd* being expressed throughout life in zebrafish, and the successful generation of a loss-of-function mutation in the *acmsd* gene, adult *acmsd*^*-/-*^ zebrafish were viable, fertile, did not develop overt morphological abnormalities, and had a similar life span to their wildtype siblings (assessed up to 25mpf). Since *acmsd* displayed strong liver expression in the larval zebrafish, we hypothesised that *acmsd*^*-/-*^ larvae may demonstrate abnormal liver development. However, the liver size of 5dpf *acmsd*^*-/-*^ larvae was comparable to that of *acmsd*^*+/+*^ zebrafish (Fig. 4), suggesting that *acmsd* deficiency does not affect liver development during the larval stages. Liver size was also assessed in larvae obtained from a homozygous incross to rule out the potential contribution of maternally-derived Acmsd. This had no effect on the results (data not included).

We next characterised *acmsd*^*-/-*^ for phenotypes relevant to PD which are altered in other zebrafish models of the disease, including changes in spontaneous movement, dopaminergic neuron quantification, microglial activation, and susceptibility to the classical PD neurotoxin MPP+^32–34,39^.

**Fig. 5:**
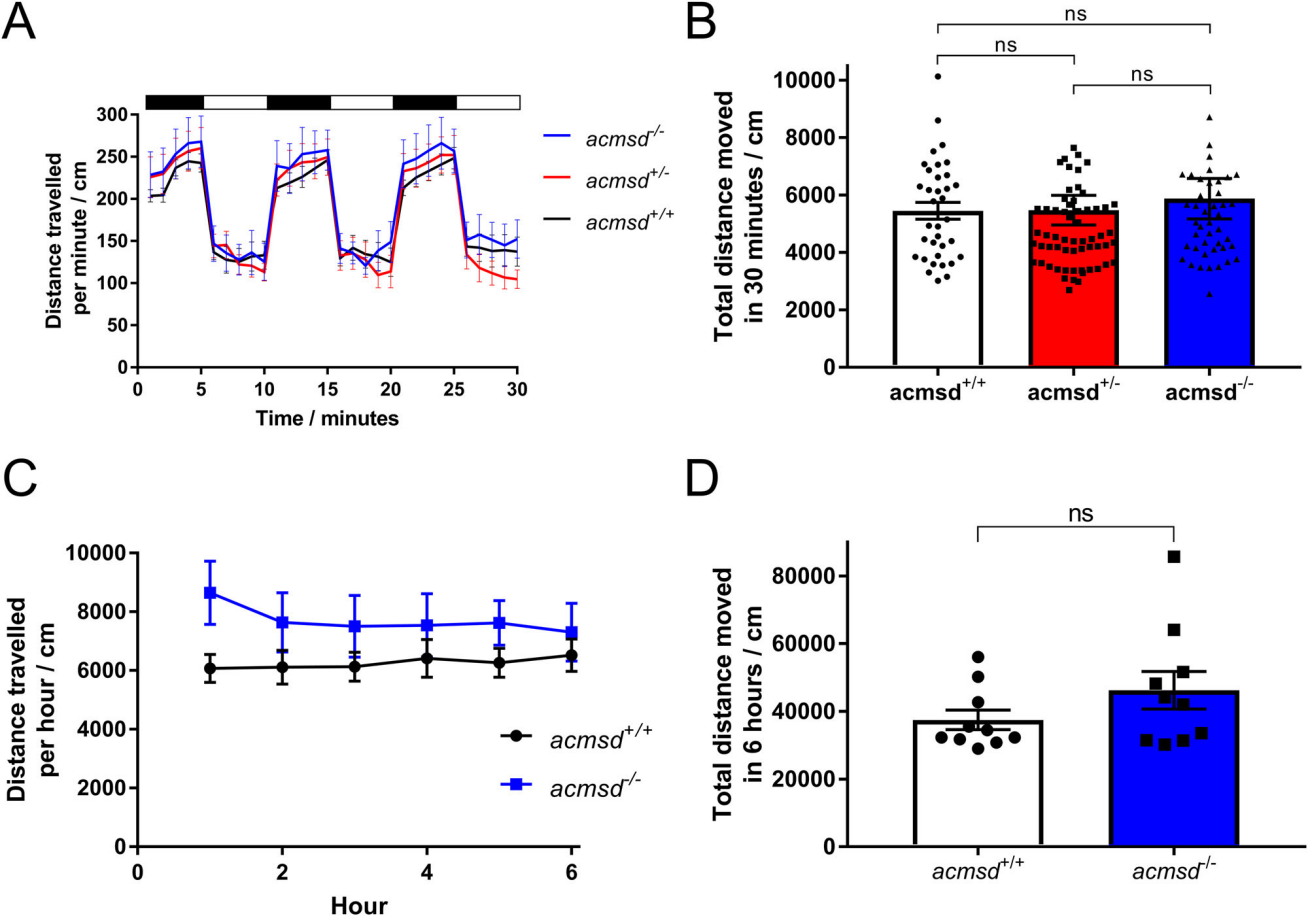
*acmsd* mutant zebrafish demonstrate normal swimming behaviour. **A** Following habituation, 5dpf larvae were exposed to alternating dark (black bar)-light (white bar) cycles. **B** Total distance travelled was indistinguishable between genotypes (Kruskal-Wallis one-way ANOVA, *p* = 0.1902). *n* = 45 *acmsd*^*-/-*^; 62 *acmsd*^*+/-*^; 35 *acmsd*^*+/+*^ from three biological replicates. **C** Following a habituation period, adult movement was recorded over 6 h at 9mpf. **D**. No difference in the total distance travelled was found between genotypes (unpaired two-tailed *t* test, *p* = 0.1796). *n* = 10 *acmsd*^*-/-*^*;* 10 *acmsd*^*+/+*^ (equal male/female ratio) taken at two separate time points. Graphs **A** + **C** show mean ± SEM. Graphs **B** + **D** show individual fish with mean ± SEM values.

Spontaneous motor activity in *acmsd*^*-/-*^ zebrafish was similar to *acmsd*^*+/+*^ at both larval and adult stages. *acmsd*^*-/-*^ larvae did not demonstrate significant alterations in swimming distance compared to their *acmsd*^*+/-*^ or *acmsd*^*+/+*^ siblings when exposed to alternating dark-light cycles (Fig. 5A, B, *p*_dark_ = 0.5567, *p*_light_ = 0.1707, *p*_combined_= 0.1902). Similarly, adult *acmsd*^*-/-*^ zebrafish did not move significantly less than their *acmsd*^*+/+*^ siblings over a 6-h period (Fig. 5C, D, *p* = 0.1796). These data suggest that a loss of Acmsd function does not have an effect on spontaneous movement in zebrafish.

**Fig. 6:**
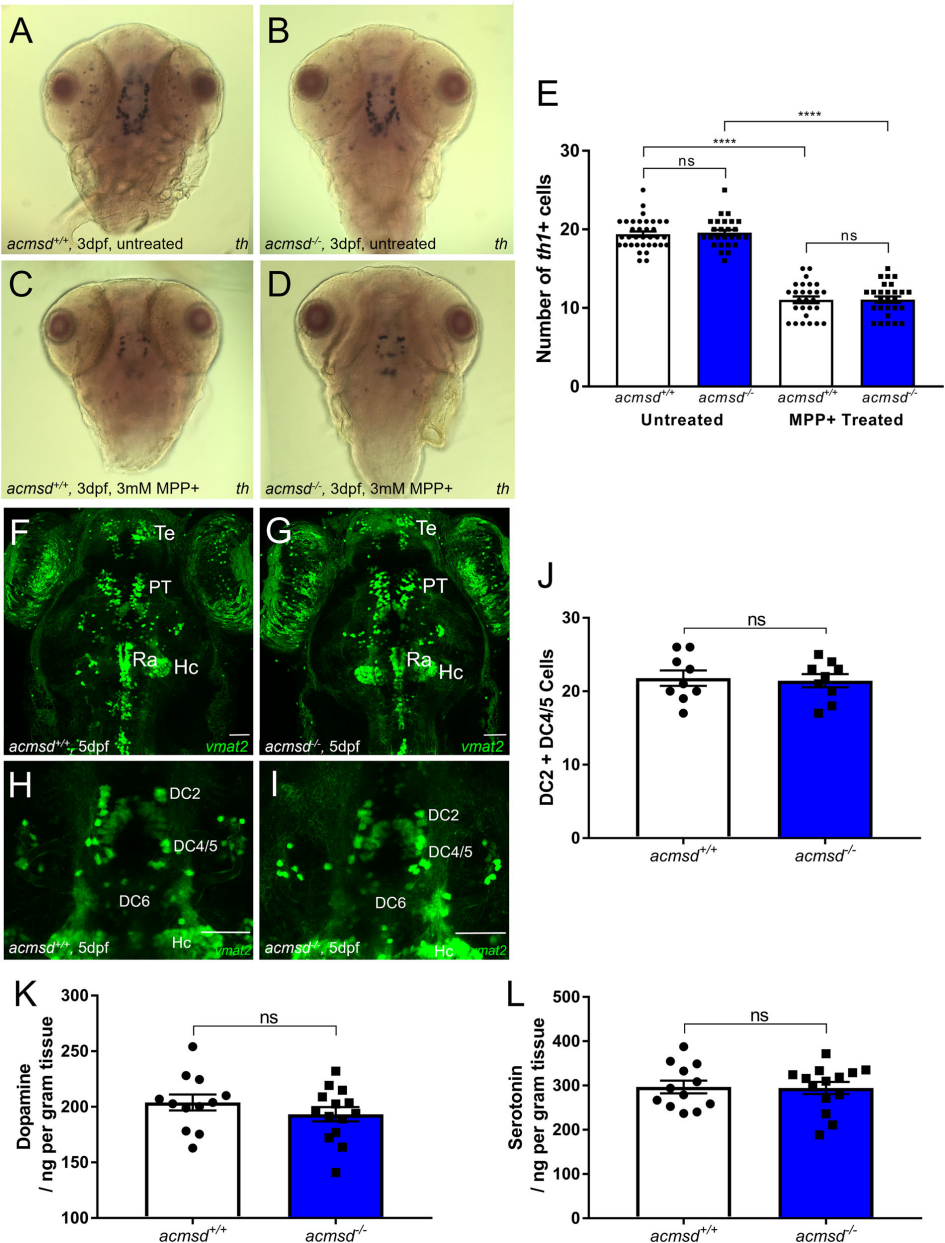
*acmsd*^*-/-*^ zebrafish retain normal dopaminergic neuron numbers and susceptibility to MPP +. **A**–**D** In situ hybridisation against *th1* showed no change in staining pattern between *acmsd*^*+/+*^ and *acmsd*^*-/-*^ zebrafish either before or after MPP+ exposure. **E** There was no difference in *th1+* neuron numbers in the ventral diencephalon between *acmsd*^*+/+*^ and *acmsd*^*-/-*^ zebrafish (*p* = 0.9866). MPP+ exposure resulted in significant reductions in *th1+* cells irrespective of genotype (43.1% in acmsd^+/+^; 43.4% in acmsd^-/-^, *p* < 0.0001). Total cell counts between groups following exposure remained indistinguishable (*p* = 0.9999). *n* = 26–35 per group, from three biological replicates. Statistics from a two-way ANOVA with Tukey’s multiple comparisons post-hoc test. Monoaminergic neurons were visualised using the *ETvmat2*:GFP transgenic line at 5dpf in the full brain (**F**, **G** dorsal view) and in the DC neuronal groups (**H**, **I**) in both *acmsd*^*+/+*^ and *acmsd*^*-/-*^ zebrafish. Scale bar = 50 µm. DC diencephalic neurons, Hc caudal hypothalamus, PT pretectal neural cluster, Ra raphe nucleus, Te telencephalic neurons. **J** No difference in the number of neuronal cell bodies in DC2 and DC4/5 was seen between *acmsd*^+/+^ and *acmsd*^*-/-*^ zebrafish (*p* = 0.8126, unpaired two-tailed *t* test). *n* = 9 per genotype, from three biological replicates.No difference in the concentrations of dopamine (**K**, *p* = 0.2808) or serotonin (**L**, *p* = 0.9079) were identified in whole 11mpf brains. Data analysed using an unpaired two-tailed *t* test. *n* = 14 *acmsd*^*-/-*^*;* 12 *acmsd*^*+/+*^, 1 brain per replicate.

We next investigated whether the loss-of-function mutation in *acmsd* resulted in a reduction in the number of ascending dopaminergic neurons in the posterior tuberculum. In situ hybridisation against *th1* revealed no significant difference in the number of dopaminergic neurons between *acmsd*^*+/+*^ and *acmsd*^*-/-*^ larvae at 3dpf (Fig. 6A–E, *p* = 0.9866). MMP+, a classical PD neurotoxin and mitochondrial complex I inhibitor, is known to reduce the number of dopaminergic neurons in larval zebrafish^33^. We hypothesised that *acmsd*^*-/-*^ larvae would be more susceptible to the effects of this neurotoxin, demonstrating a greater reduction in neuronal number compared to *acmsd*^*+/+*^ larvae. Exposure to 3 mM MPP+ from 1 to 3dpf resulted in a 43% reduction in *th1*+ cells in both *acmsd*^*+/+*^ (*p* < 0.0001) and *acmsd*^*-/-*^ larvae (*p* < 0.0001, Fig. 6C–E). The number of *th1*+ cells following MPP+ exposure was indistinguishable between genotypes (*p* = 0.9999), suggesting that *acmsd* deficiency does not alter the susceptibility of zebrafish larvae to MPP+. This experiment was also conducted on larvae obtained from a homozygous incross, similarly identifying no significant differences in *acmsd*^*-/-*^ larvae compared to wildtype controls (data not included).

We also assessed this neuronal group at a later time point (5dpf) using a different, complementary approach, namely by crossing *acmsd*^*-/-*^ zebrafish with the enhancer trap transgenic zebrafish line ETvmat2:GFP in which most monoaminergic neurons are labelled by green fluorescent protein (GFP) during embryonic development^46^. Again, there was no difference in the number of *vmat2*+ monoaminergic neurons between *acmsd*^*+/+*^ and *acmsd*^*-/-*^ larvae (Fig. 6F–J, *p* = 0.8126). Additionally, in 11mpf adult brains, the levels both dopamine and serotonin remained unchanged in *acmsd*^*-/-*^ zebrafish (Fig. 6K, L). Together, these data suggest that *acmsd* deficiency does not lead to dopaminergic neuronal cell loss in zebrafish.

### *acmsd*^*-/-*^ larvae do not develop an inflammatory phenotype

**Fig. 7:**
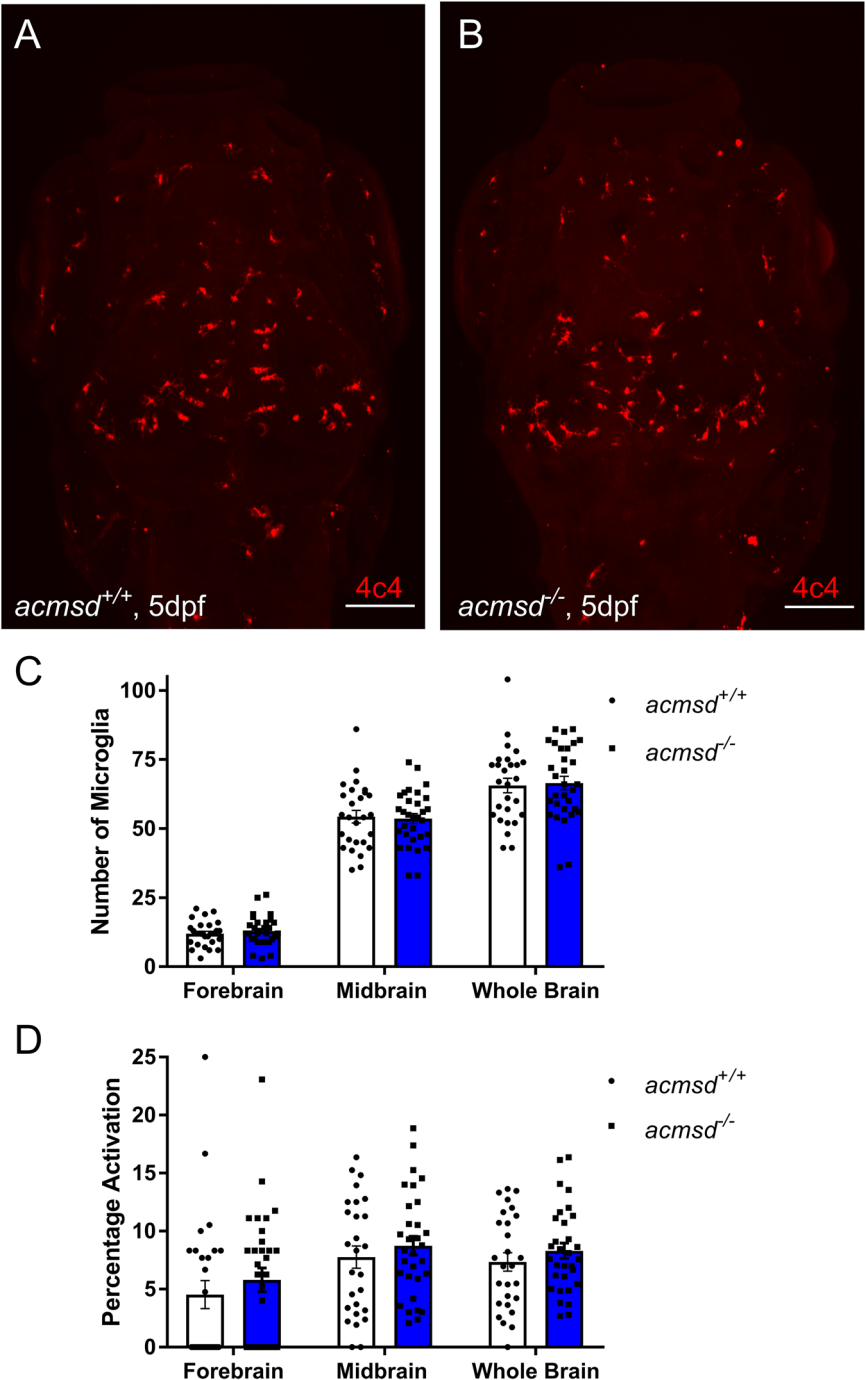
*acmsd*^*-/-*^ zebrafish retain normal numbers and activation of microglia. Representative images from wholemount immunohistochemistry against 4c4 in *acmsd*^*+/+*^ (**A**) and *acmsd*^*-/-*^ (**B**) siblings at 5dpf (scale bar = 100 µm). **C** No difference was found in the number of 4c4+ cells between *acmsd*^*+/+*^ and *acmsd*^*-/-*^ larvae in the forebrain, midbrain, or these combined (whole brain) (*p* = 0.9827 for forebrain, *p* = 0.9925 for midbrain, *p* = 0.9836 for whole brain). **D** No difference in microglial activation, represented as the percentage of amoeboid cells out of the total 4c4+ cell count, was identified between genotypes (*p* = 0.6983 for forebrain, *p* = 0.8307 for midbrain, *p* = 0.8441 for whole brain). *n* = 31 *acmsd*^*-/-*^, 27 *acmsd*^*+/+*^, from three biological replicates. Statistics analysed by two-way ANOVA with post hoc Sidak’s multiple comparisons test.

**Fig. 8:**
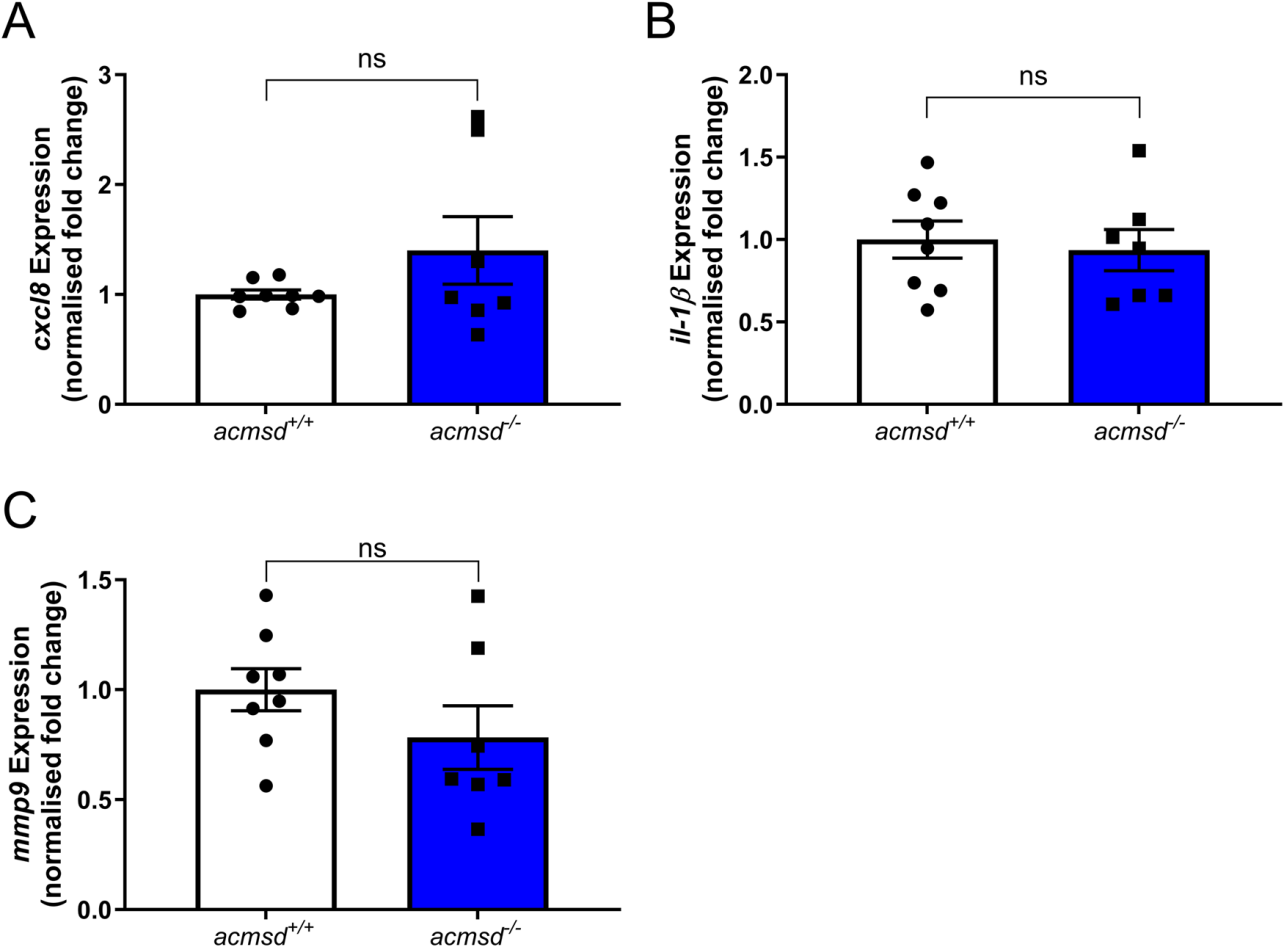
*acmsd*^*-/-*^ larvae demonstrate normal expression of three key proinflammatory mediators. qPCR data suggested that genotype had no effect on the expression of *cxcl8* (**A**, *p* = 0.9551, Mann Whitney test), *il-1β* (**B**, *p* = 0.7081, two-tailed unpaired *t* test), or *mmp9* (**C**, *p* = 0.2200, two-tailed unpaired *t* test). *n* = 7 replicates for *acmsd*^*-/-*^; 8 replicates for *acmsd*^*+/+*^ (15 larvae per replicate).

It has been postulated that ACMSD deficiency may contribute to the pathogenesis of PD through proinflammatory mechanisms^47^. Activated microglia are more numerous in both PD patient brains^48–50^ and zebrafish models of PD^33,34^. However, the number of microglia in *acmsd*^*-/-*^ larval brains was similar to the number identified in their *acmsd*^*+/+*^ siblings at 5dpf (*p* = 0.9836, Fig. 7A–C). The percentage of activated cells was also unchanged (*p* = 0.8441, Fig. 7D). qPCR showed that expression of the proinflammatory cytokines *cxcl8*, *il-1β,* and *mmp9* was also indistinguishable between *acmsd*^*-/-*^ larvae and their *acmsd*^*+/+*^ siblings (Fig. 8). qPCR analysis was also conducted on larvae obtained from a homozygous incross, which similarly identified no difference in expression between *acmsd*^*-/-*^ larvae and wildtype controls (data not included).

### Metabolomics analysis revealed both increased quinolinic acid and an upregulation of kynurenine pathway enzymes

Gene duplication and compensatory upregulation of alternative pathways in CRISPR/Cas9 mutant zebrafish lines are potential confounding mechanisms in zebrafish research^51^. To demonstrate that the kynurenine pathway is indeed disrupted in our mutants, we undertook detailed metabolomic analysis of the kynurenine synthetic pathway in *acmsd*^*-/-*^ zebrafish.

**Fig. 9:**
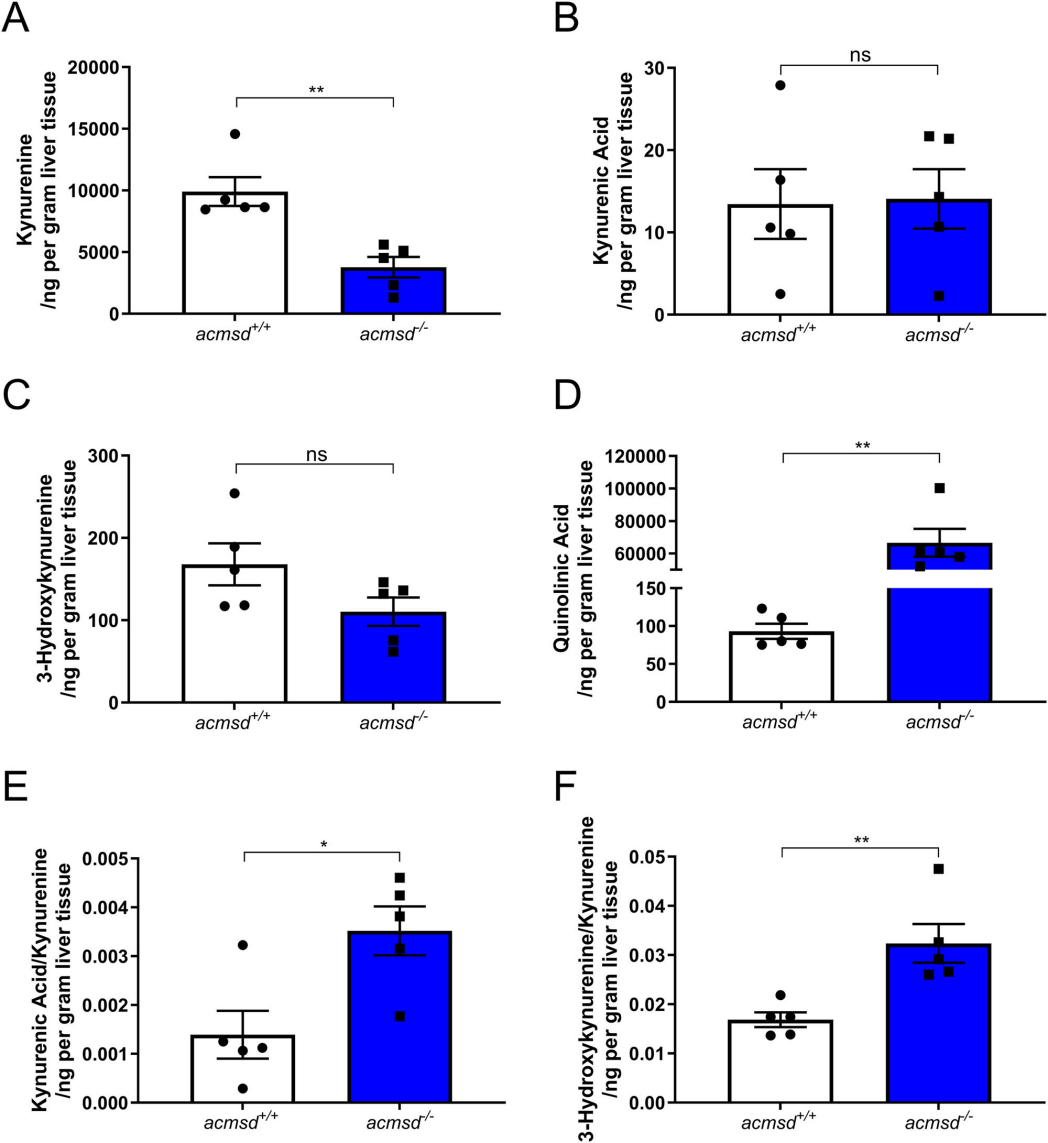
Kynurenine pathway metabolites were altered in *acmsd*^*-/-*^ adult tissues. In adult liver tissue, kynurenine was reduced in *acmsd*^*-/-*^ zebrafish (**A**, *p* = 0.0079, Mann–Whitney test), no difference was identified between genotypes in kynurenic acid (**B**, *p* = 0.9125, unpaired, two-tailed *t* test) or 3-hydroxykynurenine (**C**, *p* = 0.0986, unpaired, two-tailed *t* test), whilst quinolinic acid was increased in *acmsd*^*-/-*^ (**D**, *p* = 0.0079, Mann–Whitney test). The kynurenic acid: kynurenine ratio (**E**, *p* = 0.0161, unpaired, two-tailed *t* test) and the 3-hydroxykynurenine:kynurenine ratio (**F**, *p* = 0.0063, unpaired, two-tailed *t* test) were increased in *acmsd*^*-/-*^ fish.

As expected, metabolomic analysis confirmed a marked increase of QUIN in *acmsd*^*-/-*^ zebrafish. Whilst QUIN was undetectable in *acmsd*^*+/+*^ larvae, using the lower limit of quantification (0.42 ng/mL) we can infer that *acmsd*^*-/-*^ larvae demonstrated at least a 34,800% increase in QUIN (Table S.1). In adult zebrafish, *acmsd* deficiency resulted in an average of a 71,500% increase of QUIN in the liver (Fig. 9, Table S.1) and an 11,000% increase in the brain (Table S.1). We were unable to measure PIC as it could not be separated from its isomer, nicotinic acid. Further analysis in adult liver tissue, where metabolite concentration was high enough to enable a direct comparison between genotypes, identified the upregulation of two branches of the kynurenine pathway. The kynurenic acid:kynurenine ratio demonstrated a 153% increase in homozygous mutants (Fig. 9E), suggesting increased kynurenine aminotransferase (KAT) activity, and the 3-hydroxykynurenine:kynurenine ratio demonstrated a 92% increase (Fig. 9F), suggesting an upregulation of kynurenine-3-monooxygenase (KMO).

## Discussion

We characterised a zebrafish model of *acmsd* deficiency for a reduction in spontaneous movement, a loss of dopaminergic neurons, an increased susceptibility to MPP+, and an increase in neuroinflammation, since these phenotypes may suggest a role of *acmsd* deficiency in altering the risk of developing PD. Despite a clear and very marked effect of the genetic inactivation of *acmsd* on enzymatic activity, as observed in our metabolomic study, we did not observe a single PD relevant abnormality in *acmsd*^-/-^ larvae or adult zebrafish.

The evidence of ACMSD being involved in the pathogenesis of PD is less strong than for other putative or confirmed PD risk genes. A regulatory effect of the SNP rs6430538, located upstream of the *ACMSD* gene, and eight other SNPs found to be in linkage disequilibrium with rs6430538, on ACMSD expression has been postulated but remains unproven. Furthermore, although the existing data is not clear, there is evidence to suggest that there may also be multiple genes at this locus. Of note, the lead *ACMSD* SNP rs57891859 is actually intronic to *TMEM163*^3^, an understudied transporter protein which may be involved in lysosomal zinc handling^3,52^.

A disease-segregating mutation in the *ACMSD* gene (pTrp26Stop) has been reported in a Spanish family with cortical myoclonus, epilepsy, and parkinsonism. Only one of the six affected family members had parkinsonism, a then 53-year-old woman who first developed postural tremor of both hands at the age of 17 and generalised seizures since the age of 20. She only developed symptoms and signs of parkinsonism aged 49, including additional motor features, such as marked postural tremor and a significant memory deficit (MoCA score 19/30). A brain MRI showed abnormalities not in keeping with the diagnosis of PD, including cerebellar atrophy, high intensity brain stem signals in T2, and other changes suggestive of Wallerian degeneration^53^. Unfortunately, response to dopaminergic medication was not reported and focussed imaging of the nigrostriatal dopaminergic system, such as a DATScan—which would have confirmed the postulated loss of dopaminergic neurons—was not undertaken. A missense mutation (p.Glu298Lys) of unknown functional significance was also detected in a single late-onset patient with sporadic PD^54^. Biochemical studies of the kynurenine pathway in serum or CSF from people with sporadic PD have also given conflicting results (see Table 1).

**Table 1:** Summary of Altered Kynurenine Pathway Metabolites from People with Parkinson’s Disease

Sample and Reference	Tryptophan	Kynurenine	Kynurenic Acid	3-hydroxykynurenine	Quinolinic Acid	Kynurenic Acid/Kynurenine	3-hydroxykynurenine/Kynurenine
This Paper							
Adult zebrafish liver		↓	↔	↔	↑	↑	↑
Adult zebrafish brain		?	?	↔	↑	?	?
Whole zebrafish larvae		?	?	↔	↑	?	?
Serum							
Widner, et al.^67^	↓	↓					
Schulte, et al.^68^	↔	↔					
Han, et al. ^69^	↓	↓		↑			
Oxenkrug, et al.^70^	↓	↑	↑				
Havelund, et al.^71^	↔	↔	↓^a^	↔		↔	↔↑^a^
Sorgdrager, et al.^72^	↓	↔	↓	↔	↔		
Heilman, et al.^73^	↔	↔	↔	↑	↔		
Klatt, et al.^74^		↓		↑			
Urine							
Luan, et al.^75^	↑	↑					
CSF							
Lewitt, et al.^76^				↑			
Havelund, et al.^71^	↔	↔	↔	↔		↔	↔↑^a^
Sorgdrager, et al.^72^	↔	↔	↓	↔	↔		
Iwaoka, et al.^77^	↔	↑	↔	↑	↔	↓	↑
Heilman, et al.^73^	↔	↔	↓	↔	↔	↓	
Brain							
Ogawa, et al.^78^		↓	↓	↑		↔	

Changes in kynurenine pathway metabolites measured in human patients with Parkinson’s disease as well as those measured as part of this study. Arrows represent changes from controls.

^a^In this study, changes were only identified in patients on L-DOPA treatment who displayed signs of dyskinesia.

**Fig. 10:**
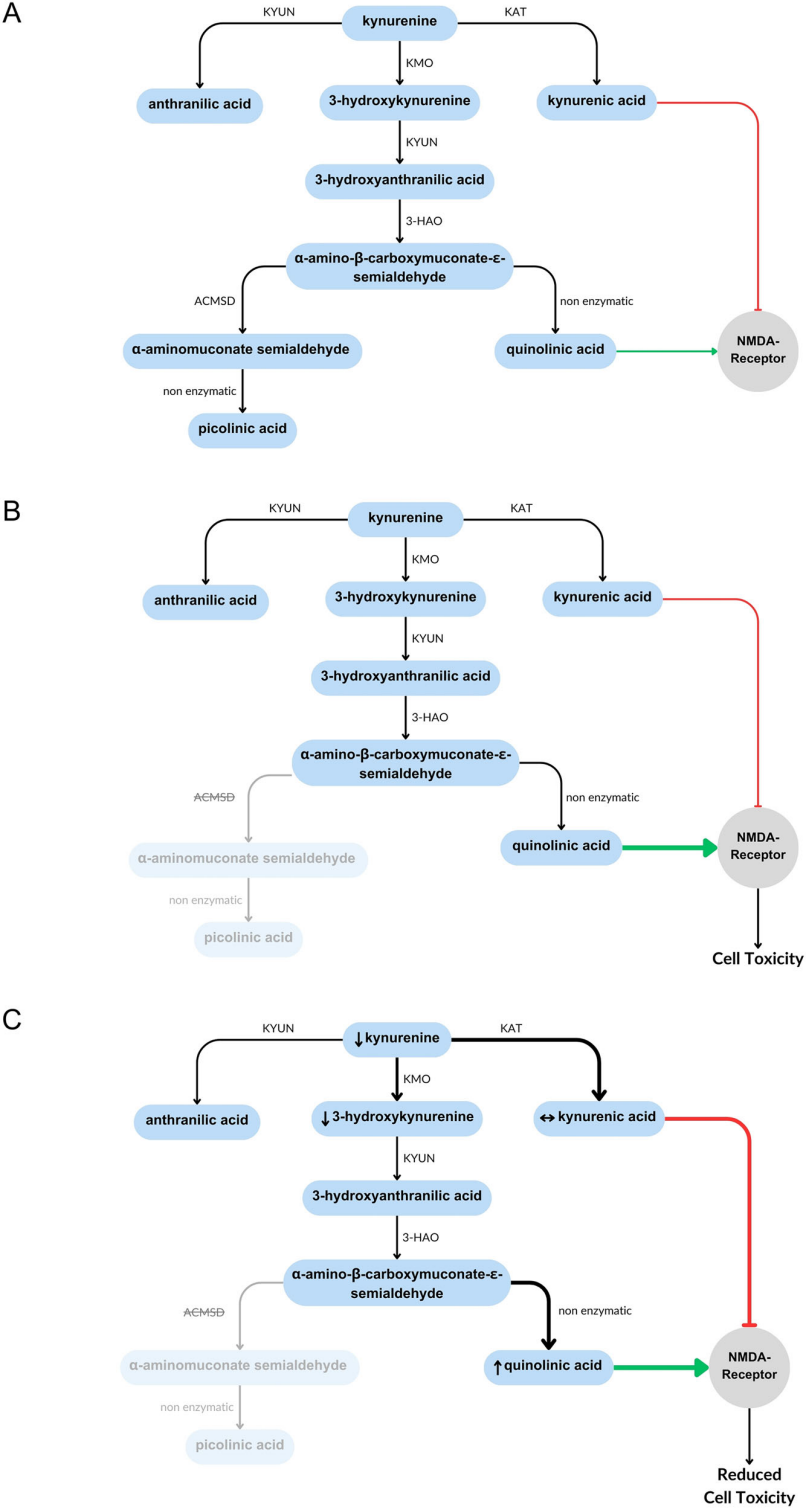
Summary of kynurenine pathway metabolite changes in *acmsd*^*-/-*^ zebrafish. **A** The basic kynurenine pathway. **B** Expected effect of reduced *acmsd* activity. **C** Results of metabolomics data from *acmsd*^*-/-*^ zebrafish. Small arrows show measured changes in metabolites in *acmsd*^*-/-*^ zebrafish compared to *acmsd*^*+/+*^. Arrow thickness represents pathway activity.

We unexpectedly identified the upregulation of two branches of the kynurenine pathway (Fig. 10). The neuroprotective pathway (kynurenic acid production) was increased to a greater extent than the canonical pathway, suggesting a compensatory upregulation of kynurenic acid, an NMDAR antagonist. This may protect against QUIN-induced toxicity and explain the absence of a phenotype in *acmsd*^*-/-*^ zebrafish.

QUIN was historically thought to only activate NMDARs with NR2A/NR2B subunits^13^, which are less widespread in the zebrafish CNS than in humans^55^. However, this selectivity has since been questioned^56^ and QUIN-induced toxicity has been demonstrated to act via non-NMDAR pathways^15–21,24–31^. Kynurenic acid, an NMDAR antagonist, also offers protection from QUIN-induced toxicity via NMDAR-independent mechanisms^24,57^. Conditioned media from QUIN-activated microglia can induce neuronal death whilst the inhibition of microglial activation reduces QUIN-induced toxicity^23^, suggesting the proinflammatory effect of QUIN may play a larger role in neuronal death than excitotoxicity.

Zebrafish are sensitive to QUIN; exogenous exposure in larvae results in stunted growth, cardiac effects, and seizures^58,59^, and in adults, direct injection into the telencephalon causes cell death and microglial recruitment^60^. Therefore, high QUIN concentrations, such as those seen throughout life in our mutant, would likely cause measurable effects in the absence of additional protective mechanisms. This further supports the hypothesis that the increased activity of the neuroprotective branch of the kynurenine pathway may be protecting *acmsd*^*-/-*^ zebrafish against toxic levels of QUIN.

It is important to note the limitations of the genetic zebrafish model, in particular that genetic compensation in CRISPR-Cas9 mutants can mask the expected functional effect of particular gene defects. However, the absence of any *acmsd* paralogues reduces this likelihood significantly in our *acmsd* mutant zebrafish line. Direct genetic compensation also appears implausible due to the observed marked increase in QUIN in both *acmsd*^-/-^ larvae and adults. We did, however, see a clear functional compensation, namely the increased activity of KAT. It is not clear whether this occurs at the pre- or post- translational level, and it would be interesting to see whether this gene is expressed at an increased level in *acmsd*^*-/-*^ zebrafish. Future experiments could include KAT morpholino injection into *acmsd*^*-/-*^ mutants to reveal a more significant role for Acmsd deficiency in zebrafish.

In summary, *acmsd* deficiency does not result in a PD-relevant phenotype in zebrafish. The results of our study, the limited genetic evidence, and the conflicting data from biochemical studies in sporadic PD patient tissue emphasise the need for further clarification of the possible role of ACMSD as a possible PD risk gene and of the importance of the kynurenine pathway in the pathogenesis of PD.

## Methods

### Zebrafish husbandry

Adult zebrafish were maintained at 28 °C in the Biological Services Aquarium at the University of Sheffield and subject to a light-dark cycle of 14 and 10 h, respectively. Embryos were obtained by pair mating and maintained in E3 media with methylene blue. 0.003% phenylthiourea [fluorochem] was used to prevent pigmentation and 4.2% tricaine used for anaesthesia. Regulated procedures followed the UK Home Office Animals (Scientific Procedures) Act 1986 under project licence PP6422743 (Professor Oliver Bandmann). All authors complied with the ARRIVE guidelines. *acmsd* loss-of-function mutants (*acmsd*^*-/-*^, allele code sh619) were created using the CRISPR/Cas9 system. A single guide RNA [Sigma-Aldrich] targeting 5′-ACTGCTCGCTATTTGTGCAT(CCA)-3′ in exon 6 of the *acmsd* gene was injected with tracrRNA and Cas9 [NEB] to single-cell stage zebrafish embryos. The line was genotyped using the following primers 5′-CCCCAGAGCTGTTTCCTGTC-3′ and 5′-CCGTGAGCAAAGCAGACCTT-3′ [Integrated DNA Technologies] as described previously^61,62^ and maintained as heterozygous colonies in the AB background. All experiments were conducted on zebrafish obtained from a heterozygous incross, unless stated otherwise.

### Quantitative polymerase chain reaction (qPCR)

RNA was extracted from homogenised adult organs or whole larvae using TRIzol Reagent [Invitrogen]. RNA concentrations were determined using the NanoDrop 1000 Spectrophotometer [Thermo Scientific] and cDNA created using the Verso cDNA Synthesis Kit [Thermo Scientific]. Transcript levels were quantified using a CFX96 Touch Real-Time PCR Detection System [BioRad] and Brilliant III Ultra-Fast SYBR Green qPCR Master Mix [Agilent Technologies]. *rps29* served as a reference gene to which expression was normalised and data were analysed using the delta-delta Ct method. Primers are outlined in Table 2. These primers were also used for reverse transcription-PCR (RT-PCR).

**Table 2:** qPCR/RT-PCR Primers Used in This Study

Gene	Forward Primer	Reverse Primer	Reference
*acmsd*	ATCCAAGAGAACTGCTGGGATC	TAACCCAACGAAGCGCTTTG	Designed in house
*il-1β*	TGCGGGCAATATGAAGTCA	TTCGCCATGAGCATGTCC	López Nadal, et al.^79^.
*mmp9*	ACGGCATTGCTGACAT	TAGCGGGTTTGAATGG	López Nadal, et al.^79^.
*cxcl-8a*	TGTTTTCCTGGCATTTCTGACC	TTTACAGTGTGGGCTTGGAGGG	López Nadal, et al.^79^.
*rps29*	TTTGCTCAAACCGTCACGGA	ACTCGTTTAATCCAGCTTGACG	Bower, et al.^80^.

Table of primers used in qPCR and RT-PCR experiments described in this manuscript

### Movement analysis

5dpf larvae obtained via a heterozygous incross were transferred to 48-well plates in 500 µL E3 and movement analysed using the ZebraBox [Viewpoint Life Sciences]. Following a 30-min habituation period, movement during alternating 5-min dark-light cycles was recorded over 30 min. Siblings were genotyped using sacrificed whole larvae. For adult movement analysis, 9mpf fish of known sex and genotype were placed in individual tanks and movement recorded for 7 h using the ViewPoint software. The first hour was omitted as a habituation period.

### MPP+ exposure

Dechorionated 3dpf larvae were exposed to 1-methyl-4-phenylpyridinium (MPP+) [Sigma] for 48 h via constant immersion in 3 mL E3 with daily media changes. Following exposure, larvae were fixed in 4% paraformaldehyde, transferred into methanol by serial dilution (30%; 50%; 70%; 100% methanol in PBSTw (PBS, 0.1% Tween-20)), and stored at −20 °C for at least 24 h.

### Wholemount in situ hybridisation (WISH)

Digoxigenin-labelled riboprobes were generated from cDNA using standard PCR and the following primers; *th1* forward 5′-AGTGCACCTGTCGGATGTTA-3′ and *th1* reverse 5′-GCGTCCACAAAGCTTTCTGA-3′; *acmsd* forward 5′- GATCCAGAGGCTCGGATTCG-3′ and *acmsd* reverse 5′-CCAGAGCATTTCCAGCAAGC-3′; *fabp10a* forward 5′-AGCTTCTCCAGAAAGCATGG-3′ and *fabp10a* reverse 5′-TCCTGATCATGGTGGTTCCT-3′. A T7 polymerase site was added to the reverse primers to generate anti-sense probes, and to the forward primers to generate sense probes. PCR products were cleaned up using the Minelute Reaction Clean-Up kit [QIAGEN] and RNA transcribed using T7 polymerase and digoxigenin-labelled NTPs at 37 °C overnight. Probes were diluted to 1 ng/µL in hybridisation buffer A and WISH performed as previously described^63^.

### Quantification of dopaminergic neurons from DC2, DC4, and DC5 subpopulations

Following WISH against *th1*, 3dpf zebrafish heads were mounted in glycerol and visualised on an AxioPlan microscope with a Plan-NEOFLUAR 20× Ph2 objective [Zeiss]. Tails were retained for genotyping. *th1*+ neurons from DC2 and DC4/5 populations, as described by Rink and Wullimann^64^, were counted blinded to genotype and treatment group. For assessment at 5dpf, monoaminergic neurons were labelled using the fluorescent vesicular monoamine transporter 2 (*vmat2*) transgenic line [allele code sh237Tg, 46]. *acmsd*^*+/-*^*;ETvmat2*:GFP zebrafish were crossed to either *acmsd*^*+/+*^ or *acmsd*^*-/-*^ zebrafish, genotyped, and imaged using an AiryScan confocal microscope [Zeiss] with a 10× objective lens.

### Microglial analysis

Immunohistochemistry against 4c4 was conducted as previously described^65^, using mouse anti-4c4 (gifted from Dr Alexander McGown, The University of Sheffield, 1:50; antibody:block) for 3 days and AlexaFluor 488 [Invitrogen] secondary antibody (1:200; antibody:PBSTw) for 3 days. Imaging and analyses were performed as previously described^33^. Cells were assigned to one of two distinct groups; active (amoeboid) or inactive (ramified with at least one visible process).

### High-performance liquid chromatography (HPLC)

Whole brains from 11mpf zebrafish of known genotype were homogenised in 300 µL homogenisation solution. Dopamine and serotonin concentrations were measured by HPLC as described earlier^66^. Measured concentrations were adjusted by tissue weight and neurotransmitters reported as tissue content (ng/g tissue).

### Kynurenine pathway metabolite analysis

Kynurenine pathway metabolite analysis was carried out by Charles River Laboratories using mass spectrometry. Five biological replicates were conducted for each experiment. Five 5dpf larvae obtained from a homozygous incross were combined and homogenised in 100 µL extraction solvent using sonication. Homogenates were centrifuged, the supernatants stored as larval extract, and metabolites reported as extract concentrations. For adult tissue analysis, brains and livers were extracted from 9mpf zebrafish obtained from a heterozygous incross and homogenised using a Precellys homogeniser in 5 µL extraction solvent per mg of tissue. Homogenates were centrifuged, the supernatants stored as extracts, and metabolites reported as tissue content (ng/g tissue).

### Statistics

All experiments were completed in at least triplicate, with each biological replicate utilising embryos from separate parental batches. Graphs and statistical analyses were conducted in GraphPad Prism 7. Graphs are shown as means ± standard error of the mean (SEM). Where relevant, normality was assessed using a Shapiro-Wilk test. Tests used are described per graph. Significance was determined as *p* < 0.05.

## Supplementary information

Supplementary Information

## Data Availability

The datasets generated during the current study are available from the corresponding author on reasonable request.

## Abbreviations

ACMS: α-amino-β-carboxymuconate-ε-semialdehyde
ACMSD: α-Amino-β-Carboxymuconate-ε-Semialdehyde Decarboxylase
AMS: α-aminomuconate semialdehyde
ANOVA: Analysis of variance
bp: Basepairs
Cas9: CRISPR-associated protein 9
cDNA: Complementary DNA
CNS: Central nervous system
COX-2: Cyclooxygenase-2
CRISPR: Clustered, Regularly-Interspaced, Short, Palindromic Repeats
CSF: Cerebral Spinal Fluid
CXCL8: C-X-C Motif Chemokine Ligand 8
DNA: Deoxyribonucleic acid
dpf: Days post fertilisation
FABP10a: Fatty acid binding protein 10a
GBA1: Glucocerebrosidase 1
GCH1: Guanosine triphosphate Cyclohydrolase 1
GFP: Green Fluorescent Protein
GWAS: Genome-wide association studies
IL-1β: Interleukin-1β
KAT: Kynurenine aminotransferase
LRRK2: Leucine-rich repeat kinase 2
Mmp9: Matrix metallopeptidase 9
MoCA: Montreal Cognitive Assessment
mpf: Months post fertilisation
MPP+: 1-methyl-4-phenylpyridinium
mRNA: Messenger RNA
NF-κB: Nuclear factor kappa B
NMDA: N-Methyl-D-aspartic acid or N-Methyl-D-aspartate
PBS: Phosphate buffered saline
PBStw: Phosphate buffered saline with 0.01% tween
PCR: Polymerase chain reaction
PD: Parkinson’s disease
PIC: Picolinic acid
PINK1: PTEN induced putative kinase 1
qPCR: Quantitative polymerase chain reaction
QUIN: Quinolinic Acid
RNA: Ribonucleic acid
RPS29: Ribosomal Protein S29
RT-PCR: Reverse Transcriptase PCR
SEM: Standard error of the mean
SNPs: Single nucleotide polymorphisms
TH1: Tyrosine Hydroxylase 1
TMEM163: Transmembrane Protein 163
TNF-α: Tumour Necrosis Factor - alpha
VMAT2: Vesicular monoamine transporter 2
WISH: Wholemount in situ hybridisation
WT: Wildtype
