# Supplementary material for: A zebrafish model of acmsd deficiency does not support a prominent role for ACMSD in Parkinson’s disease
Source: NPJ Parkinsons Dis. 2025 May 9;11:118. doi: 10.1038/s41531-025-00940-1 (PMC12064770; doi:10.1038/s41531-025-00940-1)
Supplement: Supplementary file 1 — Supplementary Information [file 41531_2025_940_MOESM1_ESM.pdf]

Table S.1. Kynurenine Pathway Metabolite Concentrations in *acmsd*$^{+/+}$ and *acmsd*$^{-/-}$ Zebrafish Tissues

Sample	Kynurenine	Kynurenic Acid	3-Hydroxykinurenine	Quinolinic Acid	Kynurenic Acid/Kynurenine	3-Hydroxykinurenine /Kynurenine
**WT Liver**						
acmsd$^{+/+}$ 1	9234	9.84	161	111	0.001066	0.017436
acmsd$^{+/+}$ 2	8641	2.50	189	75.2	0.000289	0.021872
acmsd$^{+/+}$ 3	8453	10.6	117	80.0	0.001254	0.013841
acmsd$^{+/+}$ 4	8641	27.9	118	76.4	0.003229	0.013656
acmsd$^{+/+}$ 5	14575	16.4	254	123	0.001125	0.017427
**Mutant Liver**						
acmsd$^{-/-}$ 1	5112	21.7	136	57907	0.004245	0.026604
acmsd$^{-/-}$ 2	1301	2.30	61.8	52141	0.001768	0.047502
acmsd$^{-/-}$ 3	5611	21.4	146	61834	0.003814	0.02602
acmsd$^{-/-}$ 4	2322	10.7	75.7	60915	0.004608	0.032601
acmsd$^{-/-}$ 5	4529	14.3	132	100271	0.003157	0.029146
**WT Brain**						
acmsd$^{+/+}$ 1	< LLOQ	< LLOQ	14.6	23.2		
acmsd$^{+/+}$ 2	< LLOQ	< LLOQ	10.6	18.8		
acmsd$^{+/+}$ 3	< LLOQ	< LLOQ	8.06	17.0		
acmsd$^{+/+}$ 4	< LLOQ	< LLOQ	7.39	11.1		
acmsd$^{+/+}$ 5	< LLOQ	< LLOQ	9.74	13.7		
**Mutant Brain**						
acmsd$^{-/-}$ 1	< LLOQ	< LLOQ	22.0	2030		
acmsd$^{-/-}$ 2	< LLOQ	< LLOQ	14.5	1454		
acmsd$^{-/-}$ 3	< LLOQ	< LLOQ	8.71	1220		
acmsd$^{-/-}$ 4	< LLOQ	< LLOQ	17.9	1805		
acmsd$^{-/-}$ 5	< LLOQ	< LLOQ	12.9	2858		
**WT Larvae**						
acmsd$^{+/+}$ 1	<LLOQ	<LLOQ	0.888	< LLOQ		
acmsd$^{+/+}$ 2	3.81	<LLOQ	0.751	< LLOQ		
acmsd$^{+/+}$ 3	2.89	<LLOQ	0.742	< LLOQ		
acmsd$^{+/+}$ 4	2.12	<LLOQ	0.565	< LLOQ		
acmsd$^{+/+}$ 5	<LLOQ	<LLOQ	0.502	< LLOQ		
**Mutant Larvae**						
acmsd$^{-/-}$ 1	<LLOQ	<LLOQ	0.478	91.9		
acmsd$^{-/-}$ 2	<LLOQ	<LLOQ	0.552	129		
acmsd$^{-/-}$ 3	<LLOQ	<LLOQ	0.300	197		
acmsd$^{-/-}$ 4	3.31	<LLOQ	0.913	133		
acmsd$^{-/-}$ 5	2.31	<LLOQ	0.565	183		

Table outlining the measured concentrations of kynurenine pathway metabolites from liver tissue or whole brains extracted from 9mpf zebrafish or 5dpf whole larvae (5 fish per biological replicate, combined from a single larval clutch). Adult values are given as ng/gram tissue. Larval values are given as ng/mL extract. LLOQ, lower limit of quantification.
